# A pilot randomized controlled trial of the shogi-assisted cognitive behavioral therapy (S-CBT) preventive stress management program

**DOI:** 10.1186/s13030-021-00229-8

**Published:** 2022-01-04

**Authors:** Hirokazu Furukawa, Shota Noda, Chiho Kitashima, Manami Omine, Takumi Fukumoto, Hitomi Ono, Aya Ohara, Mutsuhiro Nakao

**Affiliations:** 1 Graduate School of Education, Naruto University of Education, 748 Nakashima, Takashima, Naruto-cho, Naruto, Tokushima, 772-8502 Japan; 2 Faculty of Human Sciences, Musashino University, 3-3-3 Ariake, Koutouku, Tokyo, 135-8181 Japan; 3 Japan Society for The Promotion of Science, 5-3-1 Kojimachi, Chiyodaku, Tokyo, 102-0083 Japan; 4 Graduate School of System Design and Management, Keio University, 4-1-1 Hiyoshi, Kohoku-ku, Yokohama, Kanagawa 223-8526 Japan; 5 TAOKA Mental Health Center, 2-7-9 Joutou-cho, Tokushima, Tokushima 770-0862 Japan; 6 Aichi Health Management Center, 2-14-7 Higashisakura, Higashi-ku, Nagoya, Aichi 461-0005 Japan; 7 Kumamoto Rehabilitation Hospital, 760 Magate, Kikuyoumachi, Kikuchi, Kumamoto, 869-1106 Japan; 8 Hidamari Kokoro Clinic, 3-2-4-4 Nishiki, Naka-ku, Nagoya, Aichi 460-0003 Japan; 9 Department of Psychosomatic Medicine, School of Medicine, International University of Health and Welfare, 4-3 Kozunomo, Narita-shi, Chiba, 286-8686 Japan

**Keywords:** CBT, Preventive intervention, Board game

## Abstract

**Background:**

Shogi is a traditional board game in Japan. A preventive stress management program based on Shogi-assisted cognitive behavioral therapy (S-CBT) was applied in the Japanese municipality of Kakogawa City. The study aimed to develop an S-CBT preventive stress management program for the elderly and determine its efficacy.

**Methods:**

The participants were 67 elderly men with amateur-level Shogi skills. They were randomly assigned to either the S-CBT group (*n* = 33) or the waiting-list control group (*n* = 34). The S-CBT program was conducted over six 90-min sessions. The outcome measures were recorded using K6 instrument, the Japanese version of the abbreviated Lubben Social Network Scale, five items on cognitive behavioral functioning, and subjective well-being scale.

**Results and conclusions:**

The dropout rates of the S-CBT group and waiting-list control groups were 36.4 and 44.1%, respectively. Effect sizes (Cohen’s d) and 95% confidence intervals (CIs) were calculated for each group. Domains that changed immediately after the S-CBT intervention were problem-solving skills, self-reinforcement, and negative automatic thoughts. Future research should promote mental and physical health through the design of intervention programs using familiar materials.

**Trial registration:**

University Hospital Medical Information Network (UMIN CTR) UMIN000036003.

## Introduction

The nationwide health-promoting “Health Japan 21” campaign is a priority in Japan. Specifically, self-care has been emphasized for mental health problems [1]. A preventive stress management program is one in which subjects can acquire various strategies to cope with stressors [2]. However, effective stress management programs are not currently being implemented in Japan [3]. “Health Japan 21” is also necessary to maintain and promote physical and mental health according to regional characteristics. To develop an effective strategy for mental health, it is important to establish a stress management program that promotes self-care while being sensitive to regional characteristics.

All age groups are eligible for mental health promotion. Specifically, in Japan, where the population is rapidly aging, it is important to support the mental and physical health of the elderly, including their life functions [4]. Recent studies on the mental health of the elderly people have indicated that psychological distress leads to deterioration of physical functioning [5]. Thus, it is important to establish an effective stress management program for the elderly. The effectiveness of cognitive-behavioral programs aimed at cognitive reconstructing of negative thoughts, and the acquisition of strategies for coping with stress, has been demonstrated in the elderly [6]. Wuthrich et al. [6] implemented a group cognitive-behavioral program in a randomized controlled trial of an elderly population with anxiety and depression. They showed that anxiety and depressive symptoms improved significantly compared to the control condition. The effectiveness of cognitive-behavioral programs for treating psychological distress in the elderly has been confirmed in both Europe and the United States, but no such study has been performed in Japan. Important issues related to promoting mental health in Japan can be summarized as follows: (i) a preventive stress management program to promote the implementation of self-care while considering regional characteristics; (ii) it is necessary to consider the effect of cognitive-behavioral programs on mental health.

Kakogawa Shi, in the Hyogo Prefecture of Japan, has heavily promoted health policies based on regional characteristics. A health promotion project is being conducted by the Wellness Association public foundation in Kakogawa City, to realize “town planning where every citizen can live healthily psychologically and somatically [7].” Specifically, the town planning policy implemented by Kakogawa City utilizes “Shogi,” which is a traditional Japanese board game. However, mental health projects based on Shogi are not currently being implemented, although a role-playing game based on cognitive behavioral therapy (CBT) was recently developed [8]. CBT programs using games (e.g., board and role-playing games) can provide stress management strategies that are easy for subjects to apply. Moreover, stress management programs based on CBT can be divided into two categories: programs for high-stress (targeted) or high-risk (selective) individuals and preventive (universal) programs. In Japan, few studies examine the effectiveness of preventive programs.

In the present study, we developed a Shogi-assisted preventive stress management program for the elderly based on CBT (S-CBT) and examined its efficacy.

## Methods

### Research design

**Fig. 1:**
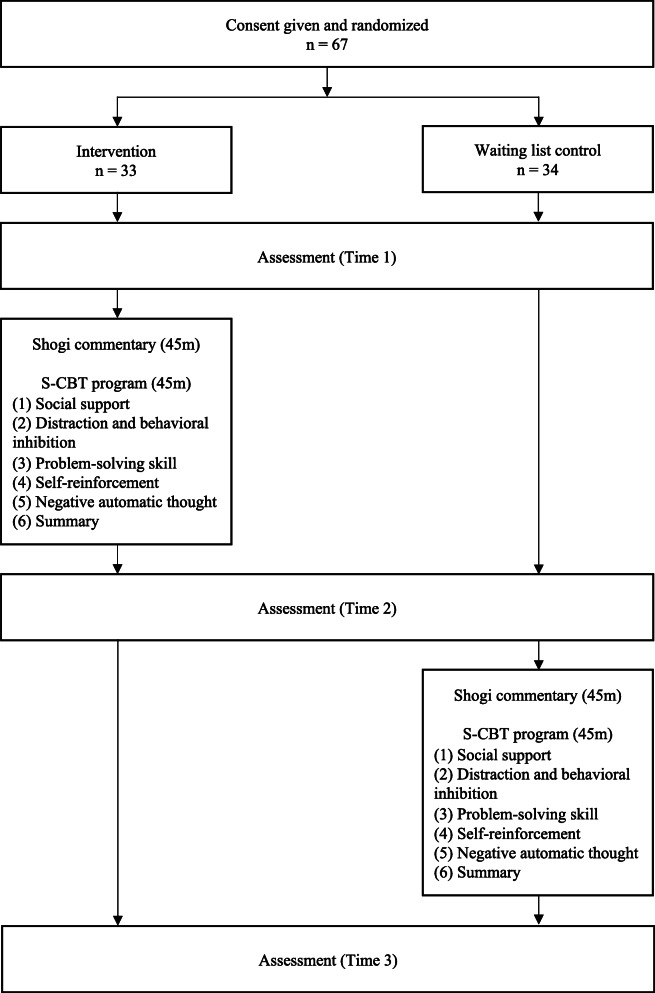
Details of participant enrollment and group assignment

A pilot randomized waiting list-controlled design was used in this study to evaluate the effects of an intervention protocol whose development was in complete. Details of participant enrollment and group assignments are shown in Fig. 1. This is an independent research paper with a different objective from that conducted by Nakao et al. [9].

### Participants

Participants were recruited from among residents living in Kakogawa-city, Hyogo, Japan using public advertisements. During the recruitment period (between September and October 2017), 67 applicants met the following inclusion criteria: (i) age > 60 years, (ii) proficiency in Shogi, and (iii) provision of written informed consent. Exclusion criteria included medical morbidities, such as dementia, which prevented completion of the test battery of self-rated questionnaires; in practice, however, no participant was excluded. Participants were assigned to the intervention group or the waiting-list control group before the baseline assessment (Time 1), conducted at the Kakogawa Shogi Plaza. Subjects allocated to the latter were instructed to visit the same location after six weeks to undergo an additional assessment (Time 2), followed by the S-CBT program. A third assessment (Time 3) was conducted for all participants after the intervention program. This study was approved by the Human Subjects Committee of Naruto University of Education and was conducted between September and December 2017.

### S-CBT program

The S-CBT program was conducted over six 90-min sessions. The intervention took about 45 min and was initiated after 45 min of instruction regarding Shogi delivered by a female player. Each group was supervised by a licensed clinical psychologist with 15 years of experience in CBT. The intervention was adapted from a general cognitive-behavioral stress management program for older adults with anxiety and depression [6]. The main components are as follows.

#### Session 1: social support

“What kinds of conversation skills are necessary for enjoying Shogi with others?” The quiz format was designed to promote the desire for social support.

#### Session 2: distraction and behavioral inhibition

We discussed improvements in behavioral strategies after delivering a lecture on “how to improve mood after losing a Shogi game and feeling sad”.

#### Session 3: problem-solving skills

Another lecture was delivered on “how to create a solution when the next move is not known in Shogi” to improve problem-solving skills.

#### Session 4: self-reinforcement

The third lecture on self-reinforcement referred to “the trick to challenge a strong opponent is stressful in Shogi” aimed to present reinforcers with the challenge of increasing their own difficult tasks.

#### Session 5: negative automatic thoughts

Finally, a lecture on cognitive distortion was delivered to increase cognitive flexibility and “to resolve the idea while considering what has been lost by Shogi”.

#### Session 6: summary of previous sessions

Sessions 1 to 5 of the program were reviewed through a quiz administered in session 6, to promote the incorporation of the cognitive and behavioral skills acquired in each session into daily life, and the ability to cope with stressors.

Participants’ cognitive-behavioral variables were compared before the start of the CBT program and after sessions. Moreover, we evaluated participants’ levels of anxiety and depressive symptoms before the start of each S-CBT session.

### Measures

#### Demographics

We obtained baseline data on participants’ age and sex.

#### The Japanese version of the K6

The K6 [10] is an instrument for measuring psychological distress that includes six items rated on a 5-point Likert-type scale, with higher scores indicating more depressive and anxiety symptoms. In the present study, the K6 was administered before the start of each S-CBT session.

#### The Japanese version of the abbreviated Lubben social network scale (LSNS-6)

The LSNS-6 [11] is used to measure social support that includes three items rated on a 6-point Likert-type scale, with higher scores indicating more social support. LSNS-6 was administered at Times 1, 2, and 3.

#### Distraction and behavioral inhibition

Distraction was indexed by a single item (“When I feel depressed, I engage in distraction activities”) rated on a 5-point Likert-type scale (1 = *never*; 5 = *usually*), with a higher score indicating more distraction activities. Distraction was assessed at Times 1, 2, and 3. Behavioral inhibition was indexed by a single item (“I am not able to do anything if I feel sad”) rated on a 5-point Likert-type scale (1 = *never*; 5 = *usually*), and a higher score indicating that the behavior was inhibited. Behavioral inhibition was assessed at Times 1, 2, and 3.

#### Problem-solving skill

Problem-solving skills were assessed by a single item (“I am confident that I can come up with many solutions to combat stress”) rated on a 5-point Likert-type scale (1 = *absolutely not*; 5 = *extremely*), with higher scores indicating better problem-solving skills. Problem-solving skills were assessed at Times 1, 2, and 3.

#### Self-reinforcement

Self-reinforcement was indexed by a single item (“I am good at praising myself”) rated on a 5-point Likert-type scale (1 = *not at all*; 5 = *very much so*), with a higher score indicating better self-reinforcement. Self-reinforcement was assessed at Times 1, 2, and 3.

#### Negative automatic thought

Negative automatic thoughts were assessed was assessed by a single item (“My unpleasant thoughts and memories persist”) rated on a 5-point Likert-type scale (1 = *never*; 5 = *usually*), with higher scores indicating more negative automatic thoughts. Negative automatic thoughts were assessed at Times 1, 2, and 3.

#### Subjective well-being scale (SWB)

The SWB [12] measures feelings and satisfaction with life using six items rated on a 5-point Likert-type scale, with higher scores indicating stronger subjective well-being. The SWB was administered at Times 1, 2, and 3.

### Data analyses

First, the participation rate in the S-CBT program was calculated. Participants who completed fewer than four sessions were considered as dropouts and thus excluded from the data analyses; the difference in dropout rates between the groups was examined.

As there are many critical views on the *p*-value in recent years [13], the effect size was calculated to clarify the intervention effect. This was a pilot study with a small sample size, and standardized effect sizes (Cohen’s d) [14], and 95% confidence intervals (95% CIs) were calculated for K6, according to the baseline (Session 1) value for each group. Similarly, Cohen’s d and 95% CIs were calculated for the LSNS-6 and SWB, and the domains of distraction and behavioral inhibition, problem-solving skills, self-reinforcement, and negative automatic thoughts, according to the Time 1 values for each group. Thus, in the intervention group, Time 2 was post-intervention, and Time 3 was the follow-up; and in the waiting list control group, Time 3 was post-intervention (Fig. 1). Cohen’s d values around 0.2, 0.5, and 0.8 are generally considered small, medium, and large, respectively; the larger the Cohen’s d value, the greater the effect [14]. Cohen’s d was considered statistically significant when the lower and upper 95% CIs did not cross zero. All analyses were performed using HAD 16.0 [15].

## Results

### Dropout rate

The dropout rates were 36.4% (12 / 33) and 44.1% (15 / 34) in the intervention and waiting-list groups, respectively. A chi-square test of the drop-out rates in both groups showed no statistical significance (*χ*^2^ = 0.41, *P* = 0.5). Thus, there was no difference in the dropout rates.

### Participant characteristics

Participant characteristics are presented in Table 1. As all subjects were male, statistical analysis of sex differences between the groups was not performed. The results of the t-test showed no significant differences between the groups for any characteristic.

**Table 1:** Demographics and baseline (Time 1) data

Variable	S-CBT (***n*** = 21)	WLC (***n*** = 19)	t	P
	M (SD), number (%)	M (SD), number (%)		
Age	72.3 (5.8)	72.2 (5.2)	0.10	0.92
Sex Male	21 (100%)	19 (100%)	–	–
Sex Female	00 (000%)	00 (000%)		
K6	4.9 (3.6)	3.9 (2.5)	0.96	0.34
LSNS-6	8.3 (3.4)	7.9 (3.3)	0.41	0.69
Distraction	3.0 (1.3)	3.5 (1.2)	1.07	0.29
Behavioral inhibition	2.1 (1.0)	2.3 (0.9)	0.57	0.57
Problem-solving skill	3.3 (1.0)	3.2 (1.1)	0.39	0.70
Self-reinforcement	2.4 (0.9)	2.9 (1.0)	1.70	0.11
Negative automatic thought	3.4 (1.2)	2.7 (1.2)	1.83	0.08
SWB	19.3 (4.9)	22.1 (3.6)	2.00	0.06

Note. *S-CBT* Shogi-assisted Cognitive Behavioral Therapy, *WLC* Waiting List Control, *M* Mean, *SD* Standard Deviation, *LSNS-6* Lubben Social Network Scale, *SWB* Subjective well-being

### K6

There was no statistically significant difference in K6 scores between the two groups in either session compared to session 1 (Table 2). Therefore, the results showed that anxiety and depression symptoms did not change with the S-CBT program.

**Table 2:** Effect of K6 score in each group

Session	S-CBT (***n*** = 21)		WLC (***n*** = 19)	
	M (SD)	d (95% CI)	M (SD)	d (95% CI)
1	4.9 (3.6)	–	3.9 (2.5)	–
2	4.5 (3.6)	−0.11 (− 0.71, 0.50)	4.2 (2.9)	0.11 (− 0.53, 0.74)
3	4.7 (4.3)	− 0.05 (− 0.65, 0.56)	2.9 (2.7)	− 0.38 (−1.02, 0.27)
4	4.5 (3.6)	− 0.11 (− 0.71, 0.50)	3.1 (2.6)	− 0.31 (− 0.95, 0.33)
5	3.8 (3.3)	− 0.32 (− 0.92, 0.30)	3.5 (2.7)	−0.15 (− 0.79, 0.49)
6	3.8 (3.8)	−0.30 (− 0.90, 0.32)	3.7 (3.1)	−0.07 (− 0.71, 0.57)

Note. *S-CBT* Shogi-assisted Cognitive Behavioral Therapy, *WLC* Waiting List Control, *M* Mean, *SD* Standard Deviation, *95% CI* 95% Confidence Interval

### Cognitive-behavioral variables and SWB

The results of the analyses of the cognitive-behavioral variables and SWB are shown in Table 3. In Table 3, Time 1, 2, and 3 are baseline, post-intervention, and follow-up for the intervention group, respectively, while Time 1 is baseline and Time 3 is post-intervention for the control group. Problem-solving skills, self-reinforcement, and negative automatic thought domains were immediately effective post-intervention, but no significant Cohen’s d values were seen for LSNS-6, behavioral inhibition, and SWB.

**Table 3:** Effect of cognitive-behavioral variables and SWB

Variable	Time	S-CBT (***n*** = 21)		WLC (***n*** = 19)	
		M (SD)	d (95% CI)	M (SD)	d (95% CI)
LSNS-6	1	8.3 (3.4)		7.9 (3.3)	
	2	7.7 (2.9)	-0.19 (-0.79, 0.42)	7.2 (3.0)	-0.22 (-0.86, 0.42)
	3	7.2 (3.1)	-0.33 (-0.94, 0.28)	7.7 (3.6)	-0.06 (-0.69, 0.58)
Distraction	1	3.0 (1.3)		3.5 (1.2)	
	2	4.0 (1.0)	0.85 (-0.21, 1.48)	3.5 (1.1)	0.00 (-0.64, 0.64)
	3	3.7 (1.3)	0.53 (-0.09, 1.14)	4.0 (0.7)	-0.50 (-0.15, 1.14)
Behavioral inhibition	1	2.1 (1.0)		2.3 (0.9)	
	2	1.9 (0.9)	-0.21 (-0.83, 0.42)	2.5 (0.8)	-0.23 (-0.41, 0.87)
	3	2.3 (1.1)	-0.19 (-0.43, 0.81)	2.0 (0.7)	-0.36 (-1.01, 0.28)
Problem-solving skill	1	3.3 (1.0)		3.2 (1.1)	
	2	4.1 (1.3)	-0.68 (-0.05, 1.30)	3.5 (0.8)	0.31 (-0.33, 0.94)
	3	3.5 (0.8)	-0.22 (-0.39, 0.82)	3.9 (0.9)	0.70 (-0.03, 1.34)
Self-reinforcement	1	2.4 (0.9)		2.9 (1.0)	
	2	3.5 (1.2)	1.02 (-0.37, 1.66)	2.8 (0.7)	-0.11 (-0.75, 0.52)
	3	2.3 (1.0)	-0.10 (-0.71, 0.50)	3.5 (0.7)	-0.68 (-0.03, 1.33)
Negative automatic thought	1	3.4 (1.2)		2.7 (1.2)	
	2	2.5 (1.3)	-0.71 (-1.33, -0.08)	3.1 (1.0)	0.35 (-0.29, 1.00)
	3	3.2 (1.3)	-0.16 (-0.76, -0.45)	2.0 (0.7)	-0.70 (-1.35, -0.04)
SWB	1	19.3 (4.9)		22.1 (3.6)	
	2	20.6 (4.2)	0.28 (-0.33, 0.89)	21.8 (3.8)	-0.08 (-0.72, 0.56)
	3	20.3 (5.0)	-0.20 (-0.41, 0.80)	21.6 (4.0)	-0.13 (-0.77, 0.51)

*Note*. *S-CBT* Shogi-assisted Cognitive Behavioral Therapy, *WLC* Waiting List Control, *M* Mean, *SD* Standard Deviation, *95% CI* 95% Confidence Interval, *LSNS-6* The Japanese version of the abbreviated Lubben Social Network Scale, *SWB* Subjective Well-Being scale

The effect sizes were medium in both the S-CBT group (d = 0.68; 95% CI: 0.05, 1.30) and WLC group (d = 0.70, 95% CI: 0.03, 1.34) for problem-solving skill. Regarding self-reinforcement, the effect size was large in the S-CBT group (d = 1.02; 95% CI 0.37, 1.66), and medium in the WLC group (d = 0.68; 95% CI 0.03, 1.33). Concerning negative automatic thoughts, medium effect sizes were found in the S-CBT group (d = − 0.71; 95% CI -1.33, − 0.08) and WLC group (d = − 0.70; 95% CI -1.35, − 0.04). However, the effect sizes at Time 3 in the S-CBT group were not significant for problem-solving skills, self-reinforcement, or negative automatic thought, unlike the effect sizes for the domains immediately after the intervention.

## Discussion

The study aimed to devise and assess an S-CBT preventive stress management program for the elderly in Kakogawa City. Few studies in Japan have examined the effect of psychological intervention programs in a randomized controlled trial setting [16], and our findings will be useful given the preliminary evidence. The effects of the six sessions of the S-CBT preventive stress management program in elderly men who were familiar with Shogi were as follows: (i) improved problem-solving skills, (ii) increased self-reinforcement behavior, and (iii) reduced negative automatic thoughts. The program had no significant effect on any other domain. Hence, although effects were found for some of the cognitive-behavioral domains, none on anxiety and depression or SWB were observed. In cognitive-behavioral preventive interventions for mental health problems, universal interventions are implemented to change cognitive-behavioral indicators, rather than improve indicators such as anxiety and depression [17]. As the study adopted a universal prevention intervention design, no effect was likely to be detected on the anxiety/depression and SWB measures because they were in the healthy range. Moreover, the level of psychological distress in our subjects was comparable to that of the community-based sample included in previous studies [12, 18]. Therefore, the degree of participants’ psychological distress may not have been sufficiently high before the S-CBT preventive program commenced, which would explain why no change occurred in certain domains. Moreover, the study examined the long-term effects by setting a follow-up period for the intervention group and found that the changes immediately after the intervention were not maintained over time. Although this study aimed for long-term effects using local characteristics and subject preferences, they were not observed. In children, it has been suggested that subject preference may be associated with long-term effects [19]; however, the factors responsible may differ between different age groups of subjects. There is a need to identify interventions that use factors other than local characteristics and subject preferences to maintain long-term effects for the elderly.

The present study had several limitations. First, a high dropout rate. A recent study using an internet-based intervention program to reduce the dropout rate reported a rate of 26% [20], which was lower than that in this study. Factors associated with dropout from cognitive-behavioral therapy programs have been found to be low motivation and/or dissatisfaction with the program, external difficulties, and participants’ feeling of improvement [21]. Although the present study did not collect data on motivation, satisfaction or external difficulties, effects related to cognitive-behavioral factors may have influenced the drop-out rate. Furthermore, adding content aimed at improving motivation could have long-term effects [22]. Future research should identify the content and delivery methods of programs that reduce dropout rates. Second, the cognitive-behavioral variables for which change was assumed, namely, social support (LSNS-6), distraction, and behavioral inhibition did not show consistent change with intervention. We identified intervention target variables in cognitive-behavioral stress management for the elderly from previous research and structured the content using a combination of validated standard procedures. In addition, all the indicators used in this study to reflect cognitive-behavioral factors were subjective indicators. Non-standardized items were used for distraction, behavioral inhibition, problem solving skill, self-reinforcement, and negative automatic thoughts. Since it is recommended to consider the use of objective measures when using non-standardized measures [23], it is possible that the present study did not reflect the changes sensitively. Future research should add objective measures to measure each indicator and identify effective procedures for changing the cognitive behavioral indicators that were not changed in this study.

## Conclusions

Our findings have several implications for community-based approaches to cognitive-behavioral prevention programs using regional characteristics and psychosomatic problems. One study reported that cognitive-behavioral stress management programs based on games have a positive effect on the ability to cope with stress [24]. These programs are effective in alleviating mental health and psychosomatic problems. However, they are not delivered to users in an optimal manner [25]. If cognitive-behavioral intervention programs based on games used materials familiar to users, the resolution of health-related problems would be more likely. Various board games are available along with Shogi for such programs. Studies seeking to promote mental and physical health through intervention programs using materials familiar to users (e.g., board games) are desirable.

## Data Availability

The obtained data and materials were used only for the present study and were available only to the researchers who participated in the study project.

## Abbreviations

CBT: cognitive behavioral therapy
S-CBT: Shogi-assisted CBT
LSNS-6: the Japanese version of the abbreviated Lubben Social Network Scale
SWB: Subjective well-being scale
95% CI: 95% confidence intervals
